# Supplementary material for: Seasonal variation in dragonfly assemblage colouration suggests a link between thermal melanism and phenology
Source: Nat Commun. 2023 Dec 19;14:8427. doi: 10.1038/s41467-023-44106-0 (PMC10730518; doi:10.1038/s41467-023-44106-0)
Supplement: Supplementary file 1 — Supplementary Information [file 41467_2023_44106_MOESM1_ESM.pdf]

Supplementary Information for:

Seasonal variation in dragonfly assemblage colouration suggests a link between thermal melanism and phenology

Roberto Novella-Fernandez
Roland Brandl
Stefan Pinkert
Dirk Zeuss
Christian Hof

Supplementary tables

Table S1: Statistical models for alternative datasets of dragonfly assemblages defined based on different combinations of parameters. Polynomial models of the spatio-phenological variation of body colour lightness (CL) using as predictors latitude and day of the year as a 4th-degree polynomial term. Polynomial models of the drivers of CL using as predictor solar radiation (rsds) as a 2nd-degree polynomial term. CL is measured as community-weighted mean of body colour lightness in standard effect size units, i.e. as deviation of observed values from those of random assemblages (see Methods).

Assemblage dataset definition	n	Spatio-phenological components of CL	Drivers of CL
resSp: 1 km<br>*resPh:* 14 days<br>*resTem*: 0 years<br>*samEff*: 4 days<br>*SamCov*: 80 %	2673	$F_{5,2667}$=406.7, R^2=0.43, p<0.001<br>Latitude: t=–11.23, p<0.001<br>Day: t=9.99, p<0.001<br>Day^2: t=–8.71, p<0.001<br>Day^3: t=7.35, p<0.001<br>Day^4: t=–6.04, p<0.001	$F_{2,2568}$=711.9, R^2=0.36, p<0.001<br>rsds: t=–11.4, p<0.001<br>$rsds^2$: t=16.56, p<0.001
resSp: 100m<br>*resPh*: 14 days<br>*resTem*: 3 years<br>*samEff*: 4 days<br>*SamCov:* 80 %	7336	$F_{5,7330}$=972.2, R^2=0.40, p<0.001<br>Latitude: t=–24.98, p<0.001<br>Day: t=16.44, p<0.001<br>Day^2: t=–14.77, p<0.001<br>Day^3: t=12.96, p<0.001<br>Day^4: t=–11.21, p<0.001	$F_{2,7147}$=1501.15, R^2=0.30, p<0.001<br>rsds: t=–13.87, p<0.001<br>$rsds^2$: t=21.46, p<0.001
resSp: 100m<br>*resPh:* 14 days<br>*resTem:* 0 years<br>*samEff:* 4 days<br>*SamCov:* 80 %	2358	$F_{5,2352}$=348.0, R^2=0.42, p<0.001<br>Latitude: t=–9.62, p<0.001<br>Day: t=9.66, p<0.001<br>Day^2: t=–8.52, p<0.001<br>Day^3: t=7.29, p<0.001<br>Day^4: t=–6.11, p<0.001	$F_{2,2287}$=613.41, R^2=0.35, p<0.001<br>rsds: t=–10.81, p<0.001<br>$rsds^2$: t=15.74, p<0.001

Note: *resSp*: Spatial resolution (m). *resPh*: Phenological resolution (days of the year). *resTem:* Temporal resolution (years). *samEf:* Sampling effort (days). *samCov:* Sampling coverage (%). See methods for details.

Table S2: Models on the phenological variation of body colour lightness (CL) of dragonfly assemblages, separately for different groups of years between 1990 and 2020. Polynomial models using CL as response and day of the year as a 4th-degree polynomial term as predictor. CL is measured as community-weighted mean of body colour lightness in standard effect size units, i.e. as deviation of observed values from those of random assemblages (see Methods).

Year group	Model
1990-1993	n=191, $F_{4,186}$=25.69, R^2=0.34, P<0.001
1994-1995	n=201, $F_{4,196}$=24.09, R^2=0.32, P<0.001
1998-1999	n=147, $F_{4,227}$=33.25, R^2=0.36, P<0.001
2000	n=193, $F_{4,188}$=34.35, R^2=0.41, P<0.001
2001	n=179, $F_{4,174}$=40.97, R^2=0.47, P<0.001
2002	n=238, $F_{4,233}$=56.55, R^2=0.48, P<0.001
2003	n=198, $F_{4,193}$=40.75, R^2=0.45, P<0.001
2004	n=252, $F_{4,247}$=32.14, R^2=0.33, P<0.001
2005	n=193, $F_{4,188}$=35.13, R^2=0.42, P<0.001
2006	n=367, $F_{4,362}$=81.03, R^2=0.47, P<0.001
2007	n=289, $F_{4,284}$=32.23, R^2=0.30, P<0.001
2008	n=361, $F_{4,356}$=74.22, R^2=0.45, P<0.001
2009	n=253, $F_{4,248}$=54.13, R^2=0.46, P<0.001
2010	n=256, $F_{4,251}$=45.39, R^2=0.41, P<0.001
2011	n=292, $F_{4,287}$=38.80, R^2=0.34, P<0.001
2012	n=498, $F_{4,493}$=53.18, R^2=0.30, P<0.001
2013	n=345, $F_{4,340}$=42.91, R^2=0.33, P<0.001
2014	n=318, $F_{4,313}$=55.17, R^2=0.41, P<0.001
2015	n=168, $F_{4,163}$=47.27, R^2=0.53, P<0.001
2016	n=439, $F_{4,434}$=96.37, R^2=0.47, P<0.001
2017	n=385, $F_{4,380}$=94.46, R^2=0.49, P<0.001
2018	n=531, $F_{4,526}$=111.5, R^2=0.45, P<0.001
2019	n=818, $F_{4,813}$=159.8, R^2=0.44, P<0.001
2020	n=594, $F_{4,589}$=78.06, R^2=0.34, P<0.001

Supplementary figures

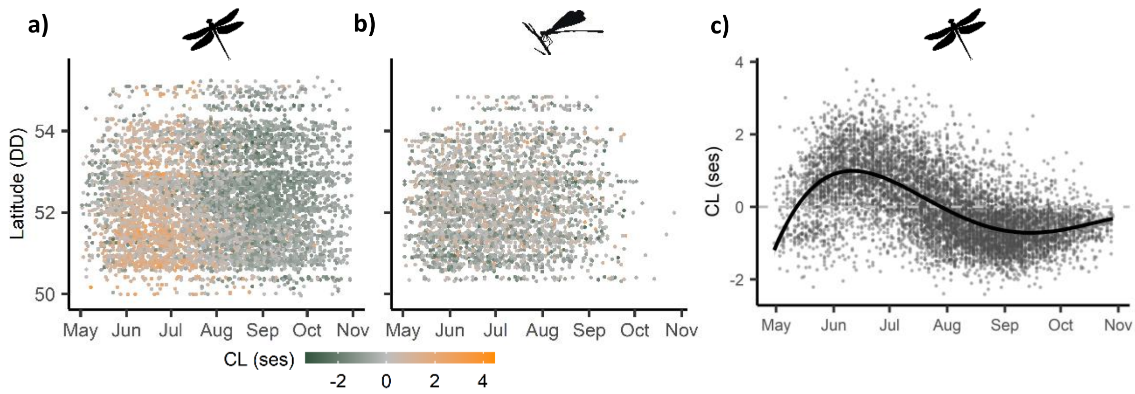

Fig. S1: Spatio-phenological variation of body colour lightness (CL) of dragonfly (a, c) and damselfly (b) assemblages based on spatially constrained null models. Variation of CL of dragonfly (a) and damselfly (b) assemblages along latitude day of the year **(c)** Residual CL variation of dragonfly assemblages along day of the year after removing the latitudinal component. Polynomial model on the spatio-phenological variation of dragonfly CL: n= 7834, $F_{5,7828}$=1132.1, R^2=0.42, *P* <0.001; Lat: t=–22.16, *P* <0.001; Day: t=17.29, *P* <0.001; Day^2: t=–15.57, *P* <0.001; Day^3: t=13.68, *P* <0.001; Day^4: t=–11.83, *P* <0.001). Spatially constrained CL indicates deviations in community-weighted mean of body colour lightness from random assemblages based on local –instead of regional– species pool. Random assemblages based on local species pools allows isolating the deviation in colour lightness corresponding to the phenological (i.e. non-spatial) replacement of species (see Methods for details on construction of spatially constrained null model).

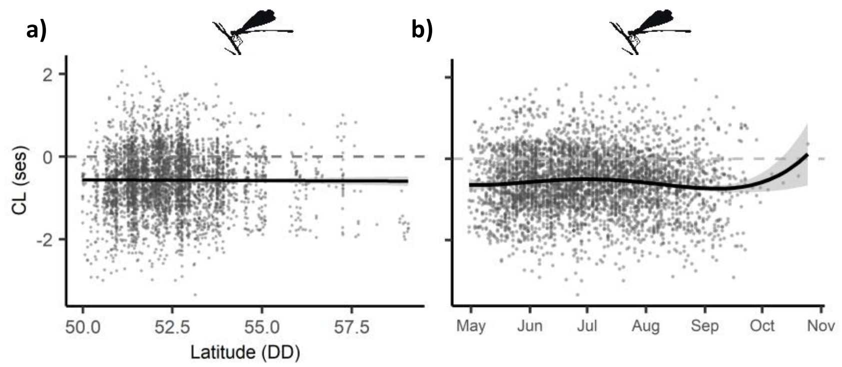

Fig. S2: Latitudinal and phenological variation of colour lightness (CL) of damselfly assemblages. CL variation of damselfly assemblages in Great Britain shows no relationship with latitude **(a)** nor day of the year **(b)**. Linear regression model: n= 4134, $F_{2,4131}$ =1.5, R^2 =0.00, *P* =0.223; Lat: t =–0.35, *P* =0.720; Day: t =–1.68, *P* =0.093.

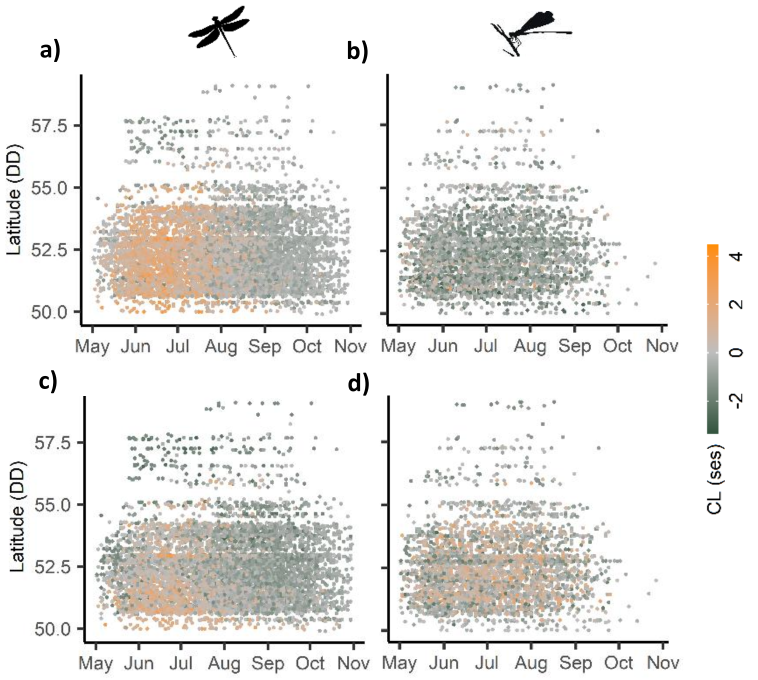

Fig. S3: Spatio-phenological patterns of body colour lightness (CL) of dragonfly (a, c) and damselfly (b, d) assemblages, separated into the phylogenetic (P) component (a, b) and the species-specific (S) component (c, d) (see Methods for additional explanations). Both P and S components of CL variation show clear signals of both latitude and day of the year for dragonflies (a, c), based on a 4th-degree polynomial model (P component: n= 8159, $F_{5,8153}$ =1196, R^2=0.42, *P*<0.001; Lat: t=–21.5, *P*<0.001; Day: t =15.72, *P* <0.001; Day^2: t =-14.05, *P* <0.001; Day^3: t =12.22; *P*<0.001; Day^4: t =–10.45, *P*<0.001; S component: n= 8159, $F_{5,8153}$=727, R^2 =0.31, *P* <0.001; Lat: t =–34.05, P<0.001; Day: t =12.07, *P*<0.001; Day^2: t =–10.57, *P* <0.001; Day^3: t =9.01, *P* <0.001; Day^4: t =–7.52, *P* <0.001). No ecologically meaningful latitudinal nor phenological patterns (i.e. very low explained variance) of CL were observed for damselflies (Linear model; n= 4134, P component: $F_{2,4131}$ =10.1, R^2 =0.00, *P* <0.001; Lat: t =4.28, *P*<0.001; Day: t =–1.61, *P* =0.107; S component: n= 4134, $F_{2,4131}$ =16.65, R^2 =0.01, *P* <0.001; Lat: t =–5.73, *P* <0.001; Day: t =–0.4, *P* =0.692).

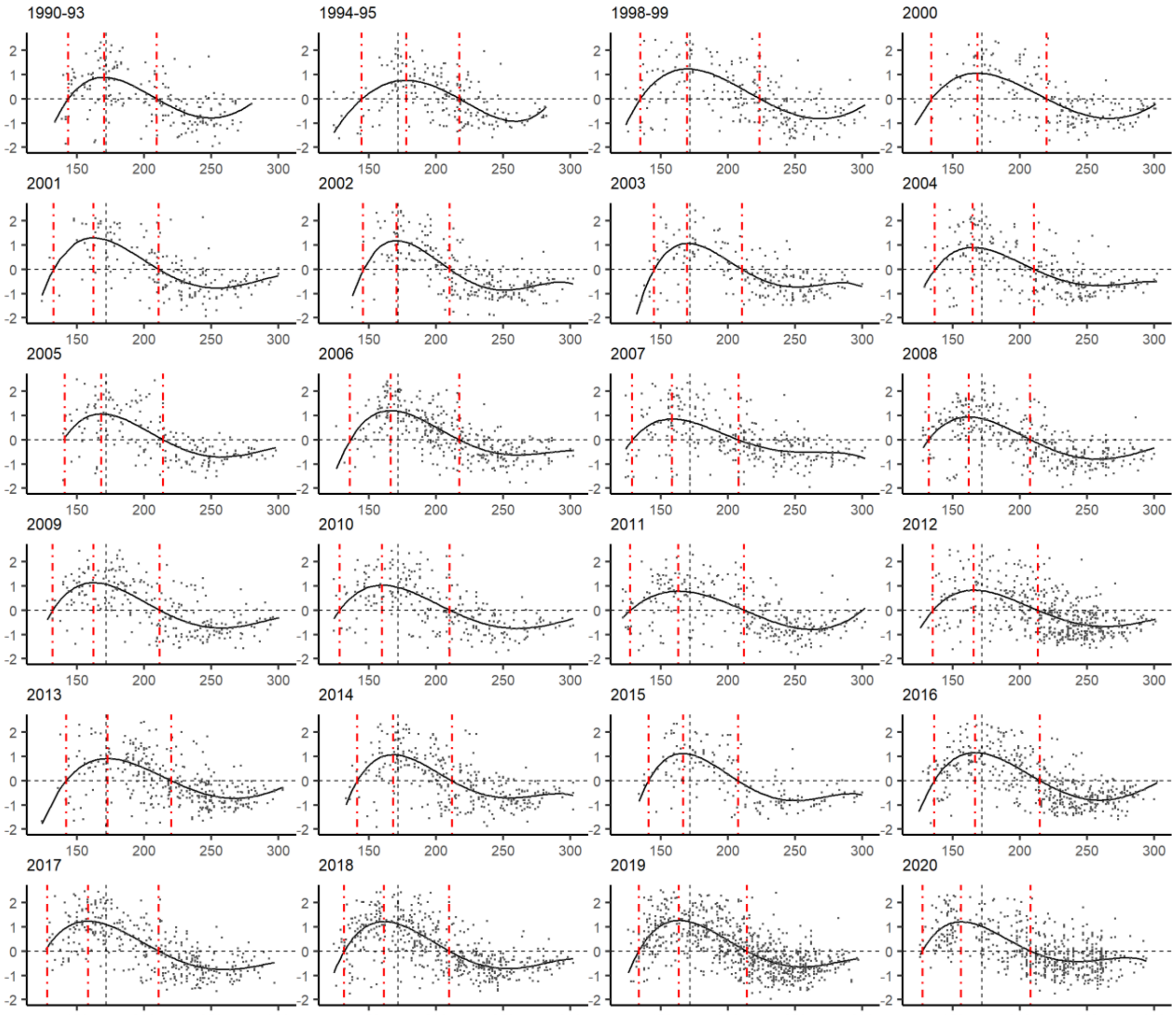

Fig. S4: Variation of the phenological pattern of body colour lightness (CL) of dragonfly assemblages over years between 1990 and 2020. Separated sequential polynomial models between 1990 and 2020 of CL depending on day of the year as a 4th-degree term. Red lines indicate, respectively from left to right: day of start of the light period (where CL is above zero). Day of CL peak. Day of end of light period (where CL is below zero). Black vertical dashed line represents summer solstice. See Methods for further details. Models´ explanatory power is shown in Table S2.

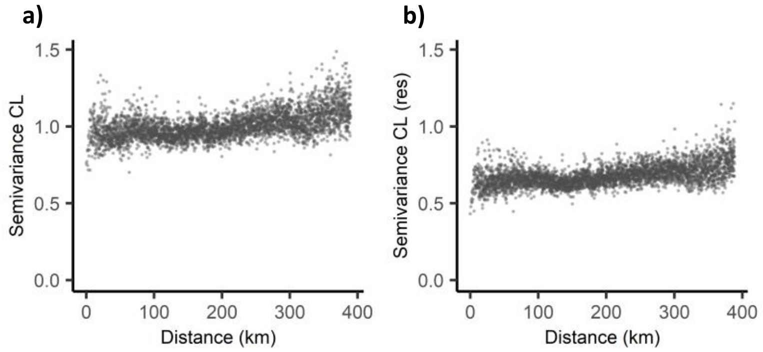

Fig. S5: Semivariograms of body colour lightness (CL) of dragonfly assemblages. (a) Semivariance of CL between pairs of assemblages at increasingly spatial distance. **(b)** Semivariance of residual CL from the polynomial model using solar radiation as a 2nd-degree term as predictors between pairs of assemblages at increasingly spatial distance. Function *variogram* in R package *gstat*[76]. A shallow increase of semivariance with distance indicates weak spatial dependence.

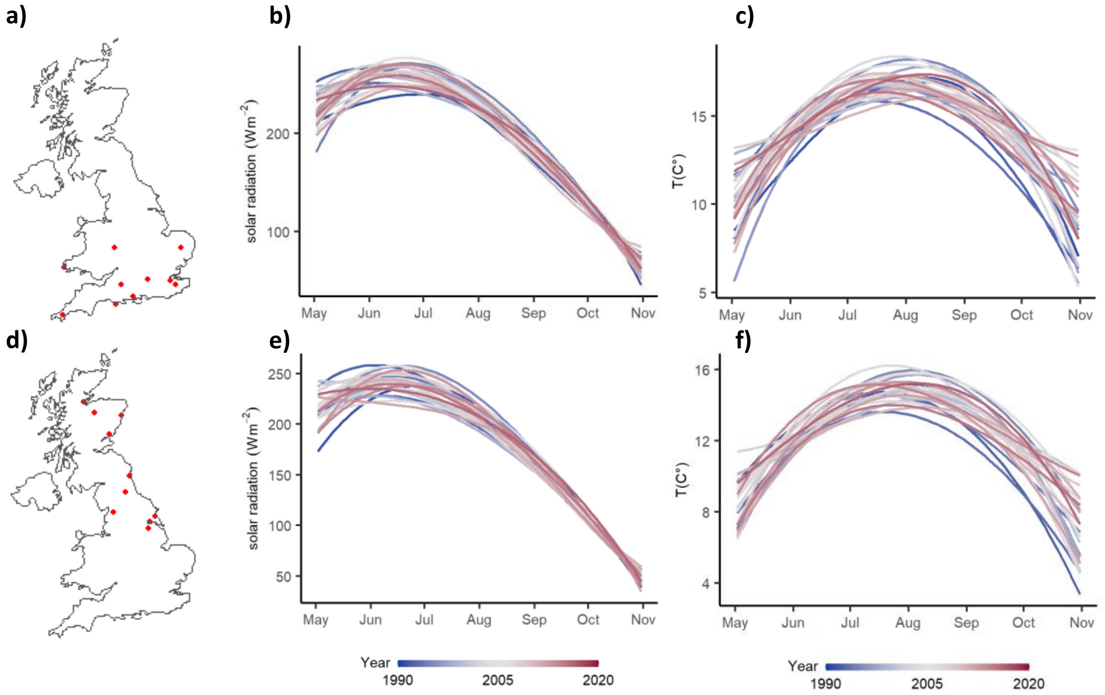

Fig. S6: Shifts in seasonal patterns of radiation (b,e) and temperature (c,f) between 1990 and 2016 in a sample of south (a,b,c) and north (d,e,f) assemblage locations (a,b) within the study region. Models of either radiaton or temperature depending on year and day of the year using polynomial terms (variable~ Day + Day^2 + Day^3+ Year) show that year did not have an effect in radiation in neither south nor north locations (South: $F_{4,44647}$ =18610, R^2=0.63, *P*<0.001; Year: t=0.01, *P*=0.99; Day: t =-118.9, *P* <0.001; Day^2: t =-119.7, *P* <0.001; Day^3: t =23.3; *P*<0.001. North: $F_{4,45745}$ =20660, R^2=0.64, *P*<0.001; Year: t=-0.33, *P*=0.74; Day: t =-123.9, *P* <0.001; Day^2: t =-113.7, *P* <0.001; Day^3: t =19.3; *P*<0.001). Conversely, year had a positive effect in temperature in both southern and northern locations (South: $F_{4,45928}$ =11170, R^2=0.49, *P*<0.001; Year: t=11.5, *P* <0.001; Day: t =-12.3, *P* <0.001; Day^2: t =-208.9, *P* <0.001; Day^3: t =0.57; *P*=0.56. North: $F_{4,45745}$ =9328, R^2=0.45, *P*<0.001; Year: t=15.4, *P* <0.001; Day: t =-11.7, *P* <0.001; Day^2: t =-189.8, *P* <0.001; Day^3: t =-1.4; *P*=0.16).
