# Supplementary material for: Seasonal variation in dragonfly assemblage colouration suggests a link between thermal melanism and phenology
Source: Nat Commun. 2023 Dec 19;14:8427. doi: 10.1038/s41467-023-44106-0 (PMC10730518; doi:10.1038/s41467-023-44106-0)
Supplement: Supplementary file 3 — Reporting Summary [file 41467_2023_44106_MOESM3_ESM.pdf]

nature portfolio

Corresponding author(s): Roberto Novella-Fernandez

Last updated by author(s): Jul 1, 2023

Reporting Summary

Nature Portfolio wishes to improve the reproducibility of the work that we publish. This form provides structure for consistency and transparency in reporting. For further information on Nature Portfolio policies, see our Editorial Policies and the Editorial Policy Checklist.

Statistics

For all statistical analyses, confirm that the following items are present in the figure legend, table legend, main text, or Methods section.

n/a	Confirmed	
☐	☒	The exact sample size (*n*) for each experimental group/condition, given as a discrete number and unit of measurement
☒	☐	A statement on whether measurements were taken from distinct samples or whether the same sample was measured repeatedly
☒	☐	The statistical test(s) used AND whether they are one- or two-sided<br>*Only common tests should be described solely by name; describe more complex techniques in the Methods section.*
☐	☒	A description of all covariates tested
☐	☒	A description of any assumptions or corrections, such as tests of normality and adjustment for multiple comparisons
☐	☒	A full description of the statistical parameters including central tendency (e.g. means) or other basic estimates (e.g. regression coefficient) AND variation (e.g. standard deviation) or associated estimates of uncertainty (e.g. confidence intervals)
☐	☒	For null hypothesis testing, the test statistic (e.g. F, t, r) with confidence intervals, effect sizes, degrees of freedom and P value noted<br>*Give P values as exact values whenever suitable.*
☒	☐	For Bayesian analysis, information on the choice of priors and Markov chain Monte Carlo settings
☒	☐	For hierarchical and complex designs, identification of the appropriate level for tests and full reporting of outcomes
☒	☐	Estimates of effect sizes (e.g. Cohen's *d*, Pearson's *r*), indicating how they were calculated

Our web collection on statistics for biologists contains articles on many of the points above.

Software and code

Policy information about availability of computer code

Data collection	The database of occurrence records was downloaded: doi:https://doi.org/10.15468/cuyjyi
Data analysis	Custom R code version 4.2.2 has been made publicly available in Zenodo https://doi.org/10.5281/zenodo.8003198.

For manuscripts utilizing custom algorithms or software that are central to the research but not yet described in published literature, software must be made available to editors and reviewers. We strongly encourage code deposition in a community repository (e.g. GitHub). See the Nature Portfolio guidelines for submitting code & software for further information.

Data

Policy information about availability of data

All manuscripts must include a data availability statement. This statement should provide the following information, where applicable:

- Accession codes, unique identifiers, or web links for publicly available datasets
- A description of any restrictions on data availability
- For clinical datasets or third party data, please ensure that the statement adheres to our policy

The raw data on odonate observations and environment are available from https://nbnatlas.org and https://data.isimip.org with the identifiers doi:https://doi.org/10.15468/cuyjyi and doi:10.48364/ISIMIP.836809.3 respectively. The processed datasets that support the findings of the study have been deposited to Zenodo https://doi.org/10.5281/zenodo.8003198.

Human research participants

Policy information about studies involving human research participants and Sex and Gender in Research.

Reporting on sex and gender	N/A
Population characteristics	N/A
Recruitment	N/A
Ethics oversight	N/A

Note that full information on the approval of the study protocol must also be provided in the manuscript.

Field-specific reporting

Please select the one below that is the best fit for your research. If you are not sure, read the appropriate sections before making your selection.

☐ Life sciences ☐ Behavioural & social sciences ☒ Ecological, evolutionary & environmental sciences

For a reference copy of the document with all sections, see nature.com/documents/nr-reporting-summary-flat.pdf

Ecological, evolutionary & environmental sciences study design

All studies must disclose on these points even when the disclosure is negative.

Study description	We downloaded a database of over one million odonate records for Great Britain. After grouping observations within fine spatio-phenological units and controlling for sampling effort by using rarefaction curves, we obtained unique datasets of 8,159 and 4,134 ecologically meaningful assemblages of dragonflies and damselflies, respectively, between May and October from 1990 to 2020
Research sample	Research sample are assemblages of dragonflies and damselflies within Great Britain between 1990 and 2020. These specific taxa were chosen to test the possible phenological operation of Thermal Melanism Hypothesis (TMH) because they has been previously demonstrated to respond strongly to TMH and show have marked phenological patterns.
Sampling strategy	We downloaded a database of all existing occurrence records of dragonflies and damselflies for Great Britain between 1990-2020. We obtained from those, 8,159 and 4,134 assemblages respectively. Sampled size recovered is enough to carry out statistical analysis.
Data collection	We downloaded a publicly available database of odonate records. Details on this data can be found at the online repository of the database: doi:https://doi.org/10.15468/cuyjyi. Details on the environmental data used can also be found also online doi:10.48364/ISIMIP.836809.3
Timing and spatial scale	Database of occurrence records aimed to range the maximum period possible. Based on availability of data we selected records between 1990 to 2020. Only high resolution records were used in space (meters) and time (specific day).
Data exclusions	No data was excluded
Reproducibility	Results can be reproduced using the R code
Randomization	Randomisation is relevant for experimental studies, but this study is observational.
Blinding	Blinding is relevant for experimental studies but this study is observational.

Did the study involve field work? ☐ Yes ☒ No

Reporting for specific materials, systems and methods

We require information from authors about some types of materials, experimental systems and methods used in many studies. Here, indicate whether each material, system or method listed is relevant to your study. If you are not sure if a list item applies to your research, read the appropriate section before selecting a response.

Materials & experimental systems

n/a	Involved in the study
☒	☐ Antibodies
☒	☐ Eukaryotic cell lines
☒	☐ Palaeontology and archaeology
☒	☐ Animals and other organisms
☒	☐ Clinical data
☒	☐ Dual use research of concern

Methods

n/a	Involved in the study
☒	☐ ChIP-seq
☒	☐ Flow cytometry
☒	☐ MRI-based neuroimaging

March 2021
